# A Transcriptional Analysis of the Genes Involved in the Ascorbic Acid Pathways Based on a Comparison of the Juice and Leaves of Navel and Anthocyanin-Rich Sweet Orange Varieties

**DOI:** 10.3390/plants10071291

**Published:** 2021-06-24

**Authors:** Paola Caruso, Maria Patrizia Russo, Marco Caruso, Mario Di Guardo, Giuseppe Russo, Simona Fabroni, Nicolina Timpanaro, Concetta Licciardello

**Affiliations:** 1 Council for Agricultural Research and Economics, Research Centre for Olive, Fruit and Citrus Crops, Corso Savoia 190, 95024 Acireale, Italy; mariapatrizia.russo@crea.gov.it (M.P.R.); marco.caruso@crea.gov.it (M.C.); giuseppe.russo@crea.gov.it (G.R.); simona.fabroni@crea.gov.it (S.F.); nicolina.timpanaro@crea.gov.it (N.T.); concetta.licciardello@crea.gov.it (C.L.); 2 Department of Agriculture, Food and Environment (Di3A), University of Catania, Via Valdisavoia 5, 95123 Catania, Italy; mario.diguardo@unict.it

**Keywords:** vitamin C, AsA metabolism, pigmented, nonpigmented, *Citrus*, citrus fruit, qRT-PCR, PCA, HPLC

## Abstract

Sweet oranges are an important source of ascorbic acid (AsA). In this study, the content of AsA in the juice and leaves of four orange clonal selections, different in terms of maturity time and the presence/absence of anthocyanins, was correlated with the transcription levels of the main genes involved in the biosynthesis, recycling, and degradation pathways. Within each variety, differences in the above pathways and the AsA amount were found between the analysed tissues. Variations were also observed at different stages of fruit development and maturation. At the beginning of fruit development, AsA accumulation was attributable to the synergic action of l-galactose and Myo-inositol, while the l-gulose pathway was predominant between the end of fruit development and the beginning of ripening. In leaves, the l-galactose pathway appeared to play a major role in AsA accumulation, even though higher GalUr isoform expression suggests a synergistic contribution of both pathways in this tissue. In juice, the trend of the AsA content may be related to the decrease in the transcription levels of the *GME*, *GDH*, *MyoOx*, and *GalUr12* genes. Newhall was the genotype that accumulated the most AsA. The difference between Newhall and the other varieties seems to be attributable to the *GLDH*, *GalUr12*, *APX2*, and *DHAR3* genes.

## 1. Introduction

Ascorbic acid (AsA) acts as a powerful antioxidant. It works as a cofactor for enzymes [1,2] and, moreover, participates in epigenetic modifications [3]. Ascorbate contributes to many signalling pathways and plays a critical role in maintaining plant redox homeostasis [4]. In plants, AsA regulates cell division and growth and is involved in in many other processes, such as flowering, senescence, and protection against environmental stresses [5,6,7,8]. AsA is essential for human health and is involved in many metabolic processes in the body: it protects from cancer, stimulates the immune system, and has many other important effects [9]. This molecule is widely used in vitamin supplements, in the food industry as a preservative to maintain plant tissues, and in derivatives (juices, vegetable pulp, etc.) with bright colours, preventing the oxidation of pigments [10,11]. Finally, a recent study [12] suggested the beneficial effects of vitamin C in patients with COronaVIrus Disease 19 (COVID-19), highlighting that intravenous ascorbate can act as an inhibitor of the pathways involved in neutrophil extracellular trap formation (NETosis) and can limit the proliferation of the inflammatory cytokine flow in the pulmonary alveoli [13]. According to Eurostat, in terms of global fruit consumption, in the last 20 years, oranges were estimated to be second only to tomatoes [1,14]. Therefore, oranges represent one of the main sources of ascorbate. Indeed, the content of ascorbic acid in citrus fruits is considered as an indicator of the quality value of fruits and fruit products [15]. The content of ascorbic acid in citrus varies according to the species and part of the fruit [16]. Very recently, Czech et al. [17] reported average values of ascorbic acid content, ranging from 50.71 ± 5.21 mg 100 g^−1^ to 30.33 ± 3.52 mg 100 g^−1^ in the pulp and in the peel of the fruit, respectively.

AsA biosynthesis takes place in all plants and the majority of animal species. In plants, the biosynthesis of AsA is more complex compared to animals, due to the probable involvement of four pathways, while in mammals AsA is synthesized through a single pathway [18,19,20].

Currently, there are thought to be four probable AsA pathways, namely l-galactose, l-gulose, galacturonate, and myo-inositol. Moreover, the total ascorbate amount, which includes two bioactive forms (reduced and oxidized), is influenced not only by its biosynthesis but also by the recycling and the catabolism pathways. l-galactose is considered the principal pathway of AsA biosynthesis. Most authors point out that, in this pathway, the key point is undoubtedly represented by the GDP mannose 3′5′-epimerase (*GME*) gene [21,22,23]. *GME* plays a dual role, catalysing two different epimerization reactions, leading to de novo AsA biosynthesis through GDP-l-galactose or GDP-l-gulose, as proposed by Wolucka and Van Montagu [21]. Moreover, many authors underlined the significant contribution of the galacturonate pathway, demonstrating its ability to increase the vitamin C level by the conversion of d-galacturonic into l-galacturonic acid, which is easily transformed to l-galactono-1,4-lactone, the direct precursor of ascorbic acid [24]. Finally, although many studies disclaim the involvement of the myo-inositol pathway [25,26,27,28], agronomic evidence supports its contribution to AsA biogenesis [24,29,30,31,32].

All these routes no doubt cooperate with each other or are active in different parts or during various development stages of plants, either for the de novo synthesis of AsA or to produce other cellular compounds. In addition to biosynthesis, the content of AsA in plants is strongly influenced by recycling and catabolism pathways [31,33,34,35,36,37], as well as transport [34,38,39].

Although the AsA content in orange fruits has already been extensively studied [40,41,42,43], few studies have been carried out on AsA biosynthesis in citrus fruit [19,31,44,45]. In a study of the AsA content in the pulp of two orange varieties, Yang and co-workers [19] suggested that the differences in AsA content were associated with differences in the expression of genes of the l-galactose pathway, as well as in the activity of enzymes involved in AsA degradation. Moreover, a comparison of the transcript levels of genes involved in AsA metabolism in the leaves, callus, flowers, and fruits of sweet orange suggested that the galacturonate pathway could be involved in AsA biosynthesis in the fruit, since some members of the d-galacturonic acid reductase (GalUR) gene family were significantly upregulated in the fruit compared to other tissues [44]. In a study performed on both the peel and pulp of fruits from two citrus species (*Citrus sinensis* and *C. unshiu*), which differ in terms of their AsA content, the l-galactose pathway appears to be predominant in both tissue types, even if the AsA concentration is regulated by complex mechanisms, in which degradation and recycling also play important roles [31]. On the other hand, the galacturonic acid pathway seems to make a relevant contribution to the AsA content of the flavedo tissue in citrus species (*C. sinensis*, *C. clementina*, *C. unshiu*, and *C. paradisi*) exposed to different light and shade regimes [45].

The objective of our work is the identification of the predominant routes and possible limiting factors of AsA metabolism in the juice and leaves of four sweet orange genotypes (two navel and two anthocyanin-rich cultivars), evaluating the accumulation of AsA and the expression of 17 genes involved in the different metabolic pathways (12 in biosynthesis, 5 in degradation and recycling). To the best of our knowledge, this is the first study that includes leaf tissue and anthocyanin-rich oranges in the assessment of gene expression in correlation with the AsA content.

## 2. Results and Discussion

**Figure 1:**
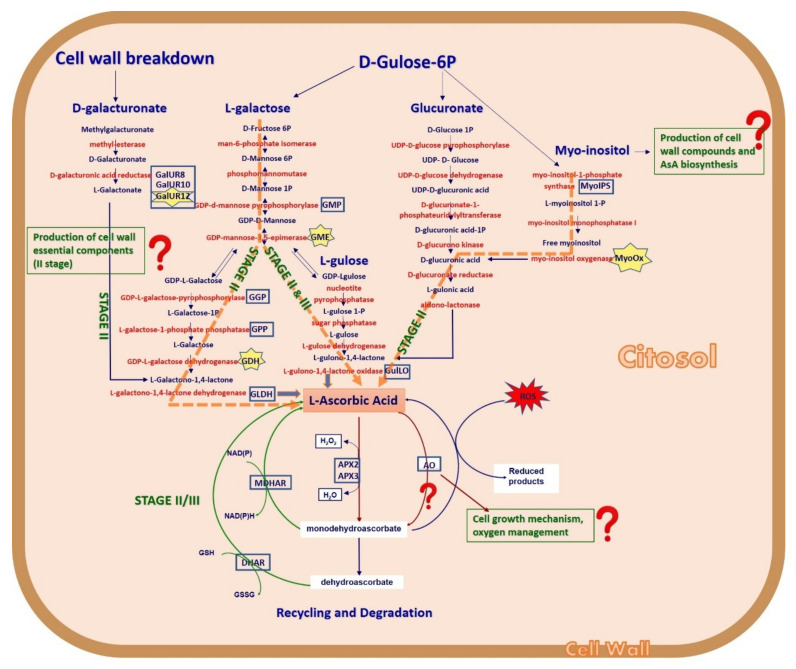
Conceptual drawing of ascorbate metabolism in citrus. This scheme represents a potential flow of reactions of the metabolism of AsA based on the data from this work and the published literature. Green capital letters indicate the periods in which the pathways occur in the biosynthesis of AsA. We hypothesize that the l-galactose and Myo-inositol pathways act during stage II through the glucuronate pathway, while l-gulose is involved in stages II and III. The recycling and degradation pathways would operate in both stages. Question marks indicate possible alternative reactions. Blue rectangles indicate genes we focused on, and genes marked with a yellow star are those we found to be crucial for the accumulation of AsA in the juice. The green arrows indicate the recycling pathway, and the red arrows indicate the degradation pathway.

The process of AsA biosynthesis in citrus fruits is not yet completely clear. In an attempt to unravel the complex metabolic pathway of ascorbic acid in citrus, we investigated the involvement of different genes and/or different pathways (Figure 1) and studied the correlation between the AsA content and transcript levels during the whole fruit development and ripening process.

Moreover, we identified possible intraspecific differences in the biosynthesis and accumulation of AsA among pigmented and nonpigmented varieties, also differing in terms of the ripening period, because most of the available information has, until now, been for comparisons between citrus species.

Finally, we envisaged possible routes leading to the production of AsA in sweet orange fruit and leaves.

### 2.1. AsA in Juice: NH the Genotype with the Highest Content

The reduced form of ascorbic acid has the greatest antioxidant activity and represents most of the oxidation states of ascorbate in plants [46]. In the present work, the transcript level was always and only correlated with the reduced form of ascorbic acid.

**Figure 2:**
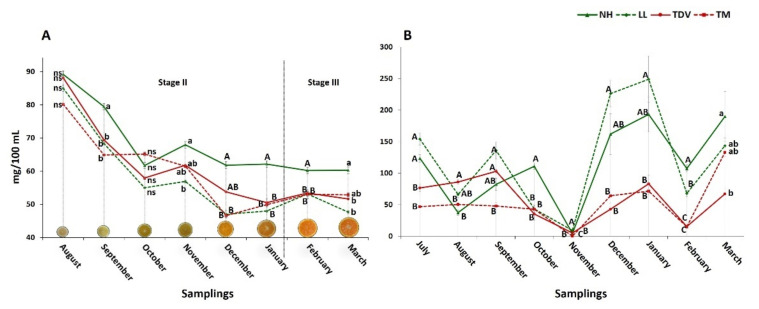
Trend of AsA concentration during sampling (August/March) in the fruit (**A**) and leaves (July/March) (**B**) of the four analysed varieties (Lanelate (LL), Tarocco Meli (TM), Newhall (NH), and Tarocco TDV (TDV)). The data are the mean ± S.E. of at least three replicates. Lowercase letters indicate *p* ≤ 0.05; uppercase letters indicate *p* ≤ 0.01. Oranges in (**A**) show the development and ripening of Newhall oranges; stage II and III are in according to Bain [47].

The AsA content in the juice was measured starting in August. The sampling in July did not allow us to extract the amount of juice needed for AsA content determination due to the small size of the fruits (3.5 × 4.5 cm on average), but it was used for the expression analysis. AsA reached the highest concentration in orange juice on approximately day 95 post-anthesis for all genotypes and then steadily decreased during fruit ripening (Figure 2A), as previously reported [19,31].

**Figure 3:**
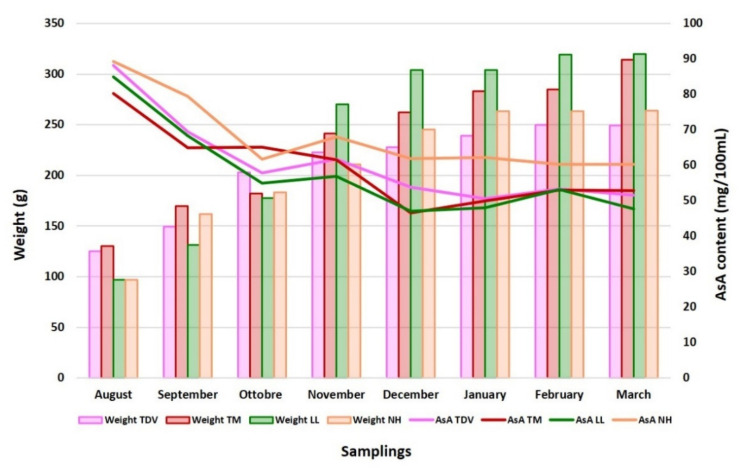
The decrease in the AsA content in relation to the stages of fruit growth and ripening in the juice of the four analysed varieties (Lanelate (LL), Tarocco Meli (TM), Newhall (NH), and Tarocco TDV (TDV)). Bars represent fruit weight and lines represent the AsA content.

The decrease in the ascorbate content during development and ripening could be due to a dilution effect caused by fruit growth, due to the accumulation of water in the fruit (Figure 3), confirming what was previously described [32,48,49]. The Pearson correlation coefficient calculated between the AsA content and fruit size and weight, evaluated during all sampling periods, had values very close to −1 for all genotypes (Table S1), indicating a perfect negative linear correlation between the compared variables. These findings reinforce the assumption that the decrease in AsA during fruit development and ripening could be due to dilution.

The accumulation of AsA was at a maximum in August (stage II), when the highest values were recorded for all genotypes (from 80.18 mg/100 mL in TM to 89.20 mg/100 mL in NH), but subsequently decreased. Despite AsA’s downward trend, a slight increase was observed in November in all varieties. NH had a higher AsA content than the other varieties throughout the sampling period, with statistically significant differences (Figure 2A) according to previous reports [19,50].

The differences found in the accumulation of AsA between the analysed varieties could also be related to the difference in the ripening period. At our experimental station, the four varieties reached commercial maturation at the following times: NH in November/December, followed by TDV in January–February, LL in March, and finally TM in April. The highest level of ascorbic acid was observed in the earliest maturing clonal selection (NH).

### 2.2. Ascorbic Acid Content in the Leaves Is Higher Than That in the Juice

The ascorbic acid content was considerably higher in the leaves (around 250 mg/100 mL) than in the juice, where the maximum value was around 90 mg/100 mL. The AsA content, especially in the leaves of navel oranges, was about 3- and 5-fold higher (in NH and LL, respectively) than in the juice (Figure 2A,B), as previously reported [51]. This could be because the leaves are more exposed to external environmental stresses than juice vesicles. Consequently, it is likely that the difference is, at least partially, attributable to the constant demand of antioxidant compounds by the leaves to contrast the effects of ROS generated by stresses, even though it is worth highlighting that a recent study underlined the role of ROS as signalling molecules regulating different responses in plants [4,6,52].

The trend of AsA accumulation in the leaves was very similar in all genotypes from November onwards. Navel genotypes accumulated a higher quantity of AsA than the pigmented ones during all sampling events, excluding September and November. In July, navel genotypes showed high values (153.91 in LL and 123.62 mg/100 mL in NH) that were statistically significant (*p* ≤ 0.01). Regarding the pigmented genotypes, the highest accumulation values were in March in TM (133.14 mg/100 mL) and in September in TDV (102.91 mg/100 mL) (Figure 2B).

The navel varieties displayed differences not only in the content but also in the AsA accumulation trend compared to the pigmented ones, especially during the summer and early autumn (Figure 2B). A strong decrease in the AsA content occurred in November for all genotypes, but this could also be due to a transfer of vitamin C from the mature leaves to the fruit, as previously reported [39].

### 2.3. Expression Analysis of Genes Involved in AsA Biosynthetic Pathways in Juice and Leaves

To investigate the involvement of AsA synthesis, recycling, and catabolism pathways in citrus juice and leaves, 17 genes catalysing pivotal metabolic stages were selected and analysed (Table S2).

#### 2.3.1. l-Galactose and l-Gulose Pathways in Juice

**Figure 4:**
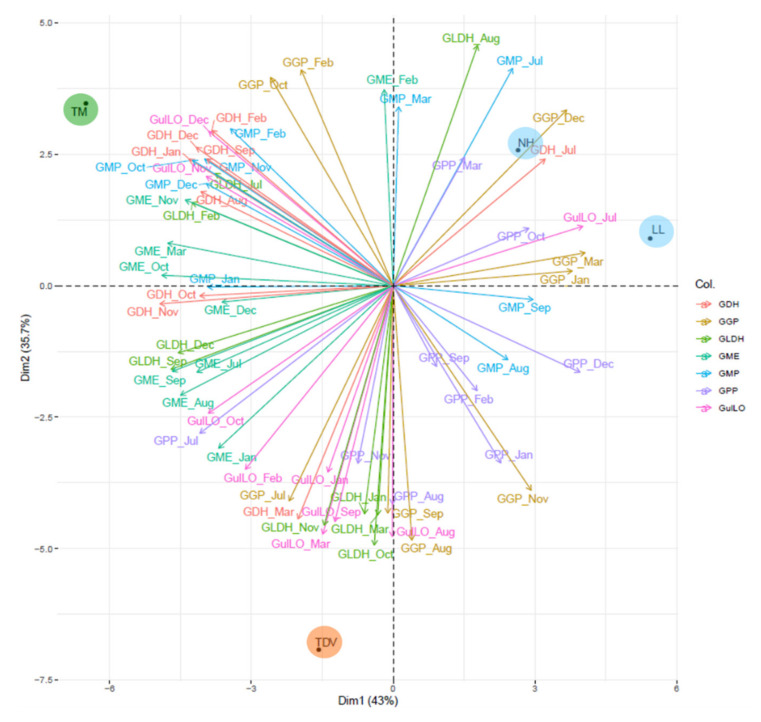
PCA of expression variability of genes involved in the l-galactose and l-gulose pathways evaluated in all sampling events (from July to March). Genes are indicated with different coloured lines. Varieties (Lanelate (LL), Tarocco Meli (TM), Newhall (NH), and Tarocco TDV (TDV)) are indicated in coloured circles.

The evaluation of these pathways by principal component analysis (PCA) explained, in the first two dimensions (Dim1 and Dim2), a total variability of 78.7%, giving insight into the clustering of navel and pigmented varieties. PCA clearly showed that most of the l-galactose pathway genes were located in the negative quadrants of Dim1, and a minority appeared in the positive quadrants. In particular, TM was located in the upper left quadrant, whereas TDV was located in the lower-left one. Both were characterized by a high expression of the *GME* gene at all time points, excluding February; the same was true for *GMP* (October, November, December, January, and February), *GDH* (November, December, January, and February), *GLDH* (September and December), *GulLO* (October, November, December, and February), and *GPP* (July). Navel genotypes (NH and LL) clustered together in the upper right quadrants (Dim1 > 0, Dim2 > 0) and showed high positive values (NH PC1 = 2.6; LL PC1 = 5.4). They were mainly characterized by a high expression of *GGP* (March, December, and January), *GDH* (July), *GulLO* (July), and *GGP* genes. Low expression (compared to the pigmented one) was instead detected for *GME* (September, August, and July), *GLDH* (September and December), and *GDH* (November) genes. Based on Dim2 (35.7% of the total variability), TM showed high similarity with the navel group, whereas TDV, which was in the negative quadrant of Dim2, was clearly separate from the navel group and was characterized by high expression of *GGP* (October and February), *GME* (February), *GulLO* (December and November), and *GDH* (February) genes (Figure 4).

##### *GME* Plays a Pivotal Role in AsA Biosynthesis

Among the genes that directly and indirectly lead to the formation of AsA in the l-galactose pathway, GDP-mannose-3,5-epimerase (*GME*) appears to play a key role, especially in pigmented genotypes, also considering the correlation that was found between the content of AsA and gene expression (Table 1), which leads us to hypothesize the involvement of this gene in the AsA decrease observed throughout the sampling period (Figure 2A). 

**Figure 5:**
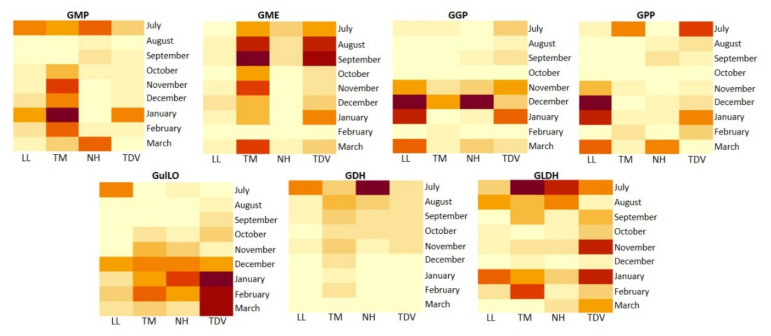
Heatmap of gene expression involved in the l-galactose and l-gulose pathways in the juice of Lanelate (LL), Tarocco Meli (TM), Newhall (NH), and Tarocco TDV (TDV) genotypes from July to March. Gene expression decreases according to the colour scale, from rust red to creamy yellow.

Furthermore, the different AsA accumulation trends, together with the differences detected in the expression level of *GME* between navel and pigmented oranges, suggest that this gene could be associated with differences between the navel and anthocyanin-rich varieties (Figure 5).

The highest values were observed for TDV and TM throughout the whole sampling period, with a peak in September. LL had a mostly constant expression level during all the sampling events and increased slightly in December, whereas NH had a decreasing trend from July to January (Figure 5). Our findings confirmed previous studies focused on the pulp of Owari Satsuma mandarins and Washington Navel oranges [31], although the authors did not include any pigmented varieties. Furthermore, the decreasing trend of *GME* gene we observed in NH is in accordance with that reported by Yang and co-workers [19] in “Egan No. 2” (Satsuma Mandarin), but our findings are in contrast with what the same authors observed in their Newhall fruits. Our data also agree with studies carried out in apples, showing that *GME* transcript levels were highly correlated with the AsA concentration during fruit development [53]. Likewise, in tomato fruits, QTL analysis showed the involvement of *GME* in controlling carbon flux and, consequently, ascorbate levels [54].

It is known that GME catalyses two distinct epimerization reactions that have, as their final product, either GDP-l-galactose or GDP-l-gulose (Figure 1), depending on the molecular form of the enzyme [55]. As reported in *Arabidopsis thaliana*, *Solanum tuberosum*, and *S. lycopersicum* [21,23,56], in citrus we assume that GDP-l-gulose could be an alternative route (via the l-gulose pathway) for the de novo biosynthesis of vitamin C.

##### *GulLO* Contributes to the AsA Content, Mostly during Ripening

It has been suggested [21] that l-gulose is oxidized to l-gulono-1,4-lactone by l-gulose dehydrogenase, which leads to the formation of ascorbic acid via l-gulono 1,4-lactone oxidase (GulLO) (Figure 1). The trigger in favour of one route instead of the other probably depends on the physiological state or the pedoclimatic conditions to which the plant is exposed.

In our study, only LL oranges showed a high *GulLO* transcript level in July (stage II), which was statistically significant compared to all the samples and to the other varieties (Figure 5 and Spreadsheet S1). The other genotypes showed the highest expression values during fruit ripening (December–February), except for TDV, which showed a maximum between January and March (Figure 5), as highlighted by the PCA (Figure 4). Therefore, we can hypothesize that the *GulLO* gene contributes to the maintenance of the AsA level, especially in pigmented citrus fruits during ripening when it is very active compared to the development stage. This assumption is also supported by the recent description of the uncommon nature of this sugar and its lack of involvement in the structural functions of the plant [1]. Thus, the activity of GulLO is thought to be due almost entirely to the constitution of de novo AsA.

##### *GMP*, *GGP*, and *GPP*

The trend and relative abundance of transcripts corresponding to GDP-mannose pyrophosphorylase (*GMP*), GDP-l-galactose-pyrophosphorylase (*GGP*), and l-galactose-1-phosphate phosphatase (*GPP*) were very similar among all the genotypes and roughly overlapped with those of *GulLO*. The highest expression of *GMP* was recorded for TM (compared with the other varieties) from October to February, with a peak in January (Figure 5). The PCA was consistent with these findings, showing a highly negative PC1 value for this genotype (Figure 4). No positive correlation was found between *GMP* transcript levels and the AsA content, as was also reported for tomato [57] and kiwi [58]. In fact, especially during the early stages of fruit development, this gene could also be involved in the formation of the noncellulosic components of the plant cell wall [34]. The *GGP* and *GPP* genes had a relatively similar trend (Figure 5). The highest expression levels were recorded for navel genotypes between the end of stage II and the beginning of stage III (from November to March), with a peak in December for *GGP* in both navel varieties and only in LL for the *GPP* gene (for both genes, the values were statistically significant compared to the pigmented genotypes) (Spreadsheet S1). Any positive correlation was found between either the expression of *GGP* and *GPP* and the AsA content (Table 1).

The high expression of *GGP* during the fall/winter (November/January) corresponded to the period in which low temperatures (10.37–12.69 °C) were recorded, confirming previous data, according to which low temperatures trigger the expression of these genes as part of AsA biosynthesis [59].

##### *GDH* and *GLDH*: Two Genes That Allow Us to Discriminate NH from Other Varieties

*GMP*, *GGP*, and *GPP* metabolic precursors enter in the flow that arrives at GDP-l-galactose dehydrogenase (*GDH*), which is located downstream of this metabolic pathway, to form AsA. Navel varieties showed a higher expression of the *GDH* gene only in July compared to the pigmented ones, particularly NH, with a transcript value of 18.90 mRNA fold increase (Figure 5). On the other hand, TM was the genotype that showed the highest values, compared to the other varieties, throughout the sampling period. In contrast, LL was the variety with the lowest transcript level. NH’s high expression value leads us to hypothesize that *GDH* may be important in the accumulation of AsA in this genotype, especially in the first stage of fruit development (July). The expression of *GDH* exhibited a decreasing trend, which correlates with the trend of the AsA content (Table 1), suggesting that this gene may be key for regulating the biosynthesis of AsA in this pathway. Our analysis showed that l-galactono-1,4-lactone dehydrogenase (*GLDH*) had the lowest expression compared to the other genes involved in the l-galactose pathway. Here, a statistically significant correlation was found only for NH and the AsA content, highlighting that *GLDH* could be involved in the varying AsA accumulation between NH and other varieties (Table 1).

#### 2.3.2. l-Galactose Pathway in the Leaves

**Figure 6:**
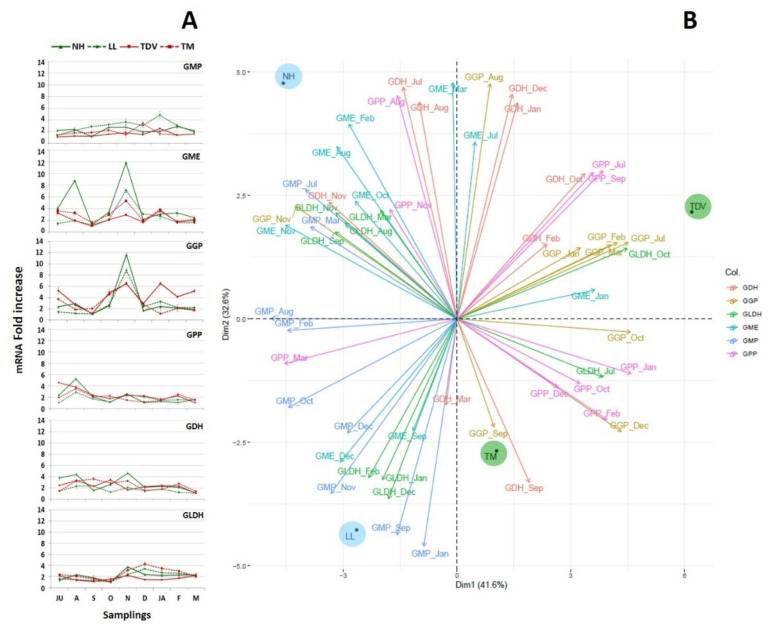
Expression analysis of genes involved in the l-galactose pathway in mature leaves of Lanelate (LL), Tarocco Meli (TM), Newhall (NH), and Tarocco TDV (TDV) genotypes during sampling from July to March. The data are the mean ± S.E. of at least three replicates (**A**). PCA of expression data of genes involved in the l-galactose pathway throughout the sampling period (**B**).

In the leaves, the expression levels of the *GMP*, *GPP*, *GDH*, and *GLDH* genes were, in general, rather low and displayed only minor differences between varieties. In contrast, *GGP* and *GME* mRNA expression showed, in all varieties, the highest values among the analysed genes. In particular, NH peaked in November for both genes (11.84 and 11.62 mRNA fold increase for *GME* and *GGP*, respectively) (Figure 6A). The high expression values of *GME* and *GGP* suggest that they could be involved in AsA metabolism, even if no positive correlation was found with the AsA content. Furthermore, the relevance of *GGP* as a pivotal gene in the regulation of AsA biosynthesis in leaves has already been documented in other species [20,60,61].

As evidenced by PCA, navel genotypes were in the negative quadrants of Dim1, which explained 41.6% of the total variability that was 74.2%. Both NH and LL were characterized by a high expression of *GME* for most of the time points. On the other hand, the pigmented genotypes were located in the positive quadrants and were characterized by a notable expression of the *GGP* and *GPP* genes in most of the months analysed (Figure 6B). Interestingly, the transcript abundance of *GGP* and *GME* was higher in navel genotypes than in pigmented ones in November (Figure 6A); we assume that a transcriptional mechanism could anticipate the peak of the AsA content, as shown in December–January (Figure 2B). By contrast, the low expression of these genes in September and October could partially justify the low AsA content recorded in the leaves in November, although a potential translocation of AsA (as mentioned above) could be assumed, as previously described [58].

#### 2.3.3. *GulLO* Expression Is Missing in Leaves of Citrus

In the leaves, it was not possible to evaluate the expression level of *GulLO*, even though several attempts were made. The sequence of *GulLO*, whose expression was evaluated in juice, was selected as a homologue of *AtGulLO5* (*At2g46740*) in sweet oranges. Considering that no expression was obtained in leaves, a further investigation was dedicated to this gene. We included the name “d-arabinono-1,4-lactone” as a keyword in the *Citrus sinensis* Annotation Project [44]. Among the three results reported by the search (*Cs3g25370.1*, *Cs7g31580.1*, and *orange1.1t00763.1*), and considering that the expression in tissues reported as RNAseq data showed a high expression in the leaves for the last two, we designed oligos for real-time PCR expression analysis for *Cs7g31580.1* and *orange1.1t00763.1*. Real-time PCR analysis reported an “undetected” result. We concluded that, in contrast to juice, *GulLO* was not expressed in leaves. We need to further evaluate these novel data. No evidence has been previously reported for the evaluation of genes involved in AsA biosynthesis in citrus leaves.

#### 2.3.4. Myo-Inositol through Glucuronate Pathway in Juice and Leaves

##### Myo-Inositol in the Juice

Myo-inositol plays a multifunctional role in plant growth, being involved in signal transduction and stress-related responses, and also being pivotal for cell wall polysaccharide synthesis [62]. Moreover, it catalyses the first step of myo-inositol catabolism, generating d-glucuronic acid (d-GlcUA), an essential sugar precursor of the plant cell wall [63] that is known to maintain the myo-inositol level needed for the synthesis of other compounds in plants [64].

The conversion of d-Gulose-6P to l-myo-inositol 1-P is catalysed by myo-inositol phosphate synthase (MyoIPS); subsequently, l-myo-inositol 1-P is converted to free myoinositol by myo-inositol monophosphatase I (Figure 1). The oxidation of free myo-inositol by myo-inositol oxygenase (MyoOx) produces GlcUA, which is a potential compound entering into the cascade that leads to AsA synthesis (Figure 1).

**Figure 7:**
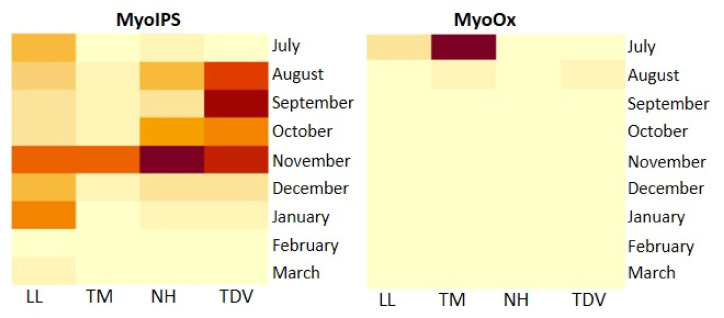
Heatmap of *MyoIPS* and *MyoOx* gene expression in the juice of Lanelate (LL), Tarocco Meli (TM), Newhall (NH), and Tarocco TDV (TDV) genotypes from July to March. Gene expression decreases according to the colour scale, from rust red to creamy yellow.

In the juice of the four sweet orange varieties, *MyoIPS* showed the highest expression in November (Figure 7).

The portion of myo-inositol not converted to d-GlcUA remains as myo-inositol 1-P and could be incorporated into a putative recycling mechanism or could be used by GGP as a substrate producing AsA through the l-galactose pathway [57].

Even if Myo-inositol is considered a minor pathway among the routes producing AsA, there are several findings illustrating that MyoOx is a key enzyme, as reported in pepper, tomato, kiwi, and strawberry [57,65,66,67,68,69]. In agreement with these authors, we found the overexpression of *MyoOx* in the early phase of fruit development (Figure 7) to be positively correlated with the content of AsA (Table 1). The high expression levels of the *MyoOx* gene, detected much more during this phase (and less later on), indicated that the myo-inositol route via d-GlcUA acid was less induced by ripening compared to the development stage, confirming the previous results [70]. Similarly, Alos et al. [31] reported relevant *MyoOx* expression, even in the peel of immature green oranges. The significant decline in *MyoOx* expression during fruit ripening could indicate that the accumulation of AsA would be attributable to the synergic action of the l-galactose and Myo-inositol pathways in the first stage of fruit development.

##### Myo-Inositol in the Leaves

**Figure 8:**
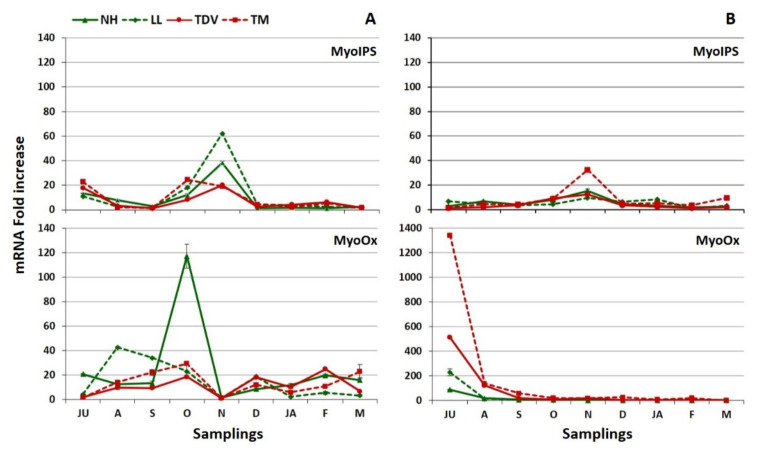
Real-time expression data of *MyoIPS* and *MyoOx* genes in the mature leaves (**A**) and juice (**B**) of Lanelate (LL), Tarocco Meli (TM), Newhall (NH), and Tarocco TDV (TDV) genotypes during sampling from July to March. The data are the mean ± S.E. of at least three replicates.

The expression level of *MyoIPS* was higher in the leaves than in the juice, especially in July, October, and November (Figure 8A,B). In July, the mRNA fold increase values of pigmented genotypes were higher compared to navel ones; in October, the maximum value was reported in TM (24.57 mRNA fold increase); in November, the transcription levels of navel genotypes were higher than those of pigmented ones. In contrast, very low expression values were registered, not only in August and September but also during winter sampling events, starting in December (Figure 8A).

The *MyoOx* expression pattern in the leaves was very different in terms of both the trend and the expression level compared to the juice (Figure 8A,B). The lowest expression was observed in November (1.0 to 2.0 mRNA fold increase), while in juice it decreased, starting in October, especially for the navel genotypes (Figure 8B). The expression values observed in July and August were considerably lower than those in the juice (Figure 8A,B). The maximum value (117.35 mRNA fold increase) was reported in October for the NH genotype. This is the first time that the *MyoIPS* gene has been investigated in citrus. Our data highlighted its potential involvement, together with *MyoOx*, in the leaves, in accordance with previous studies [29,71]. However, other studies assert that *MyoOx* contributes minimally to the AsA content in the leaf tissue [25,28].

#### 2.3.5. d-Galacturonate Pathway

**Figure 9:**
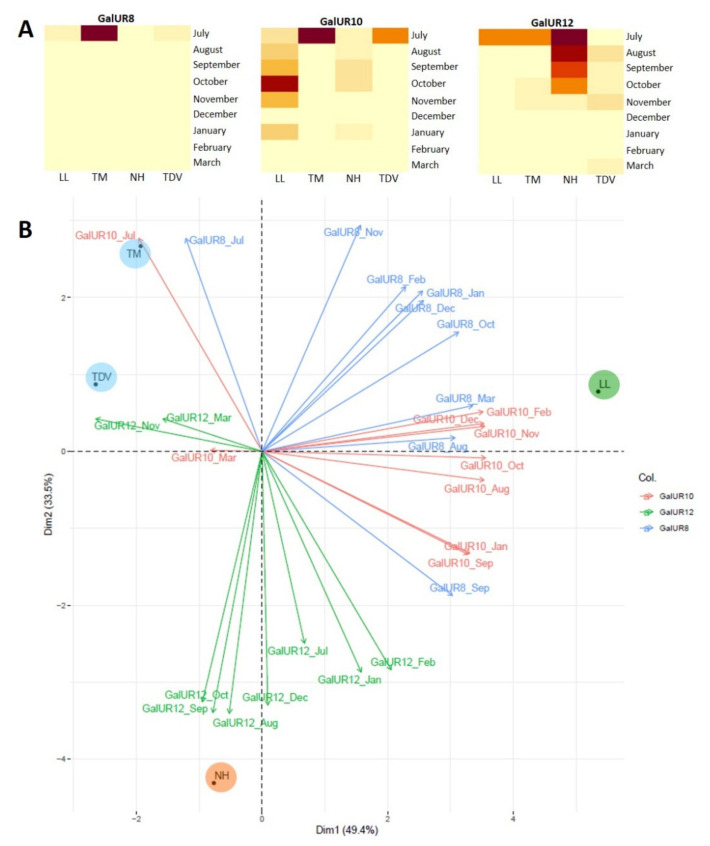
Heatmap (**A**) and PCA (**B**) of the gene expression data of three GalUR isoforms in the juice of Lanelate (LL), Tarocco Meli (TM), Newhall (NH), and Tarocco TDV (TDV). In the heatmap, gene expression decreases according to the colour scale, from rust red to creamy yellow. In the PCA, each isoform is a different colour. Varieties are indicated in coloured circles.

The three d-galacturonic acid reductase isoforms (*GalUR8*, *GalUR10*, and *GalUR12*) analysed in this work had different expression patterns, in agreement with what was observed by Xu et al. [44]. *GalUR10* had the highest expression values for navel genotypes in almost all samples (Figure 9A); specifically, LL showed a peak of 198.08 mRNA fold increase, in October, which was statistically significant compared to all other expression values (Spreadsheet S1). Moreover, at the beginning of fruit development (July), *GalUR10* was considerably overexpressed in TM and TDV (219.57 and 124.04 mRNA fold increase, respectively) (Figure 9A).

*GalUR8* showed a peak in July for all genotypes, and the highest value was registered for TM (101.57 mRNA fold increase) (Figure 9A). A negative or no correlation was seen between the *GalUR8* or *GalUR10* transcript levels and the AsA content (Table 1), confirming evidence reported by Alos et al. [31]. The high transcript levels of the *GalUR8* and *GalUR10* isoforms, especially at the beginning of stage II, and the lack of correlation with the AsA content contribute to the indication that the two isoforms are likely involved in the production of essential cell wall components, such as pectin, typical of stage II fruit development, in agreement with previous reports on other species [72].

The expression level of *GalUR12* of NH showed the highest value compared to the other varieties, from July to October, then declined more drastically until March (Figure 9A). NH and TM were the only varieties in which *GalUR12* was correlated with the AsA content (Table 1). This correlation would suggest that *GalUR12* plays a pivotal role in the regulation of AsA accumulation in citrus juice, especially during fruit development, in accordance with previous studies [44]. The high expression of *GalUR12* in NH could also be associated with the AsA content and may help to explain the differences in AsA accumulation between varieties (Figure 2A). These data were supported by a PCA, in which the two dimensions explained the involvement of *GalUR* genes in AsA biosynthesis with a variability of 82.9%. Dim1 and Dim2 accounted for 49.4% and 33.5%, respectively, of the total variability. PCA showed that the two pigmented genotypes had similar expression patterns of *GalUR* genes, while the two navels differed considerably. The PCA clearly highlighted the high *GalUR12* expression observed in NH during the first stages of fruit development, confirming that the expression of this gene was related to the high AsA accumulation observed in NH (Figure 9B).

Overall, the expression values of the three *GalUR* isoforms were lower in the leaves than in the juice, and the highest values were recorded from November to January. The highest expression was reached for *GalUR8* in NH in July (Figure S1). Similarly, *GalUR10* and *GalUR12* showed the highest transcript levels between November and January, indicating a possible synergic contribution of the l-galactose and d-galacturonate pathways to AsA biosynthesis in citrus leaves.

#### 2.3.6. Recycling Pathway

Concerning the recycling pathway, two genes have been analysed: Monodehydroascorbate reductase (*MDHAR*) and one isoform of dehydroascorbate reductases (*DHAR3*).

**Figure 10:**
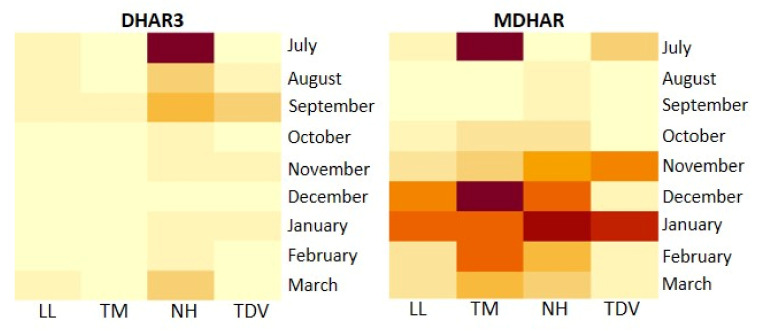
Heatmap of DHAR3 and MDHAR gene expression in the juice of Lanelate (LL), Tarocco Meli (TM), Newhall (NH), and Tarocco TDV (TDV) genotypes from July to March. Gene expression decreases according to the colour scale, from rust red to creamy yellow.

Monodehydroascorbate (MDA) can be reduced by MDHAR and converted into AsA, which is the form capable of responding to oxidizing agents. Therefore, a certain part of AsA could be produced via recycling (Figure 1), working on feedback or any other mechanism to regulate the AsA content. Indeed, MDHAR maintains the size and redox status of the AsA pool [73] and is of fundamental importance in oxidative stress tolerance [74]. Our data showed an increase in *MDHAR* expression in all genotypes between the end of development (stage II) and early ripening (November/January), with TM having the highest expression in July, and from December to March, and NH showed an upward trend of transcription that peaked in January (11.25 mRNA fold increase) (Figure 10).

The transcription levels of *DHAR3* were generally lower than those of *MDHAR* and rather stable, with slight fluctuations during the whole period, suggesting that MDA was greatly reduced and a small amount of DHA was formed. We observed some differences in terms of expression levels; NH was the highest during all sampling events, except in December (Figure 10).

Although the overexpression of *MDHAR* and *DHAR3* was recorded in NH, the positive correlation between the AsA content and NH and the TDV expression of *DHAR3* supports the role of this gene in enhancing the AsA content in the NH genotype and also in TDV, which had the second-highest AsA accumulation (Figure 2A). By contrast, no positive correlation was found between the *MDHAR* gene and AsA concentration (Table 1). Our data fit with those described by other authors, suggesting that *MDHAR* and *DHAR3* could be involved in maintaining the regular concentration of AsA in fruit when the levels decrease due of environmental reasons, such as heat or chilling stress [37,75,76].

In the leaves, *MDHAR* and *DHAR3* genes showed very similar trends for all the analysed genotypes (Figure S2A). The highest transcripts were recorded in November. The *MDHAR* expression level was slightly lower than in the juice (Figure S2A,B); *DHAR3* revealed a transcript range that was slightly higher than in the juice only in November (Figure S2A,B). The expression decrease (in the leaves) in *MDHAR* and *DHAR3* in September and October could be associated with the low AsA content detected in November in all genotypes (Figure 2B), also considering the low expression of biosynthetic *GME* and *GGP* genes in the same months (Figure 6A). On the contrary, the high expression of the same genes in November (especially for navel genotypes) could be associated with the highest AsA values recorded in the leaves in December and January (Figure 2B).

#### 2.3.7. Degradation Pathway

**Figure 11:**
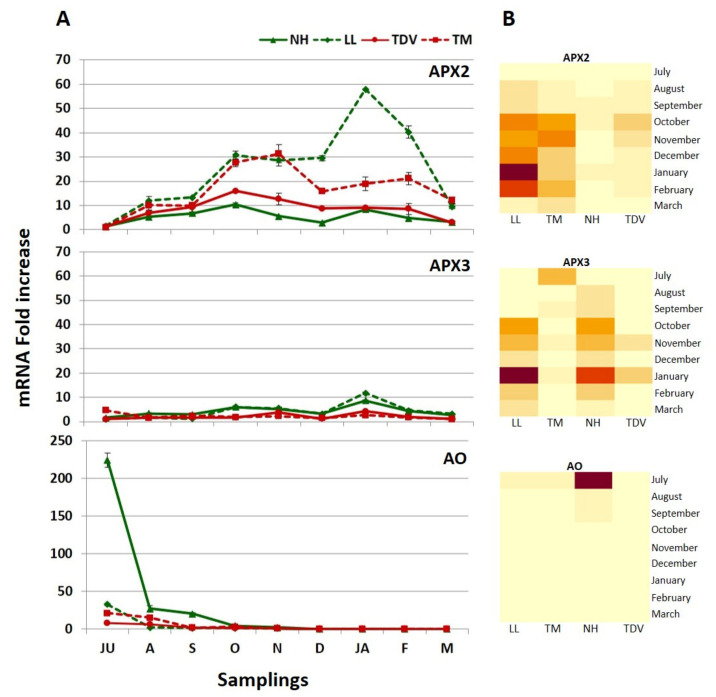
Real-time expression data (**A**) and heatmap (**B**) of the *APX2*, *APX3*, and *AO* genes involved in the degradation pathway in the juice of Lanelate (LL), Tarocco Meli (TM), Newhall (NH), and Tarocco TDV (TDV) genotypes from July to March. The data are the mean ± S.E. of at least three replicates. In the heatmap, gene expression decreases according to the colour scale, from rust red to creamy yellow.

Among the genes related to the degradation pathway, we investigated two l-ascorbate peroxidase isoforms (*APX2* and *APX3*) and ascorbate oxidase (*AO*). The partial oxidation of AsA is often catalysed by the action of APX and causes the formation of monodehydroascorbate (MDHA) (Figure 1). AsA also has an indirect action in detoxifying ROS, this being the substrate through which APX converts H_2_O_2_ to water [18] (Figure 1). Although the diversity and functions of plant APXs have been exhaustively investigated, the mechanisms by which AsA neutralizes the different ROS produced during metabolic processes in plants are still not entirely clear [77]. Among the isoforms we evaluated in our work, *APX2* had the highest expression in orange juice. The transcription profile fluctuated for some varieties (Figure 11A,B).

Starting in October until the end of the sampling period, the highest statistically significant values were displayed by LL (57.84 mRNA fold increase) and TM (31.32 mRNA fold increase) compared to NH and TDV (Figure 11A,B and Spreadsheet S1). These data support the lack of an AsA increase, observed during the sampling events. In NH, the low expression level of *APX2* could also contribute to maintaining its high concentration of AsA. Although lower than *APX2*, the expression of *APX3* showed the highest values in January, particularly in the navel genotypes (11.76 and 8.62 mRNA fold increase LL and NH, respectively) (Figure 11A,B). Furthermore, statistically significant differences were found between navel and anthocyanin-rich genotypes (Spreadsheet S1). These findings are in agreement with what was previously described in the pulp of navel oranges for the same isoforms [31]. The different *APXs* expression levels found in this work reinforce the hypothesis that AsA degradation by APXs is crucial for the regulation of the AsA content in citrus juice.

The expression levels of the *AO* gene were high during the first stage of fruit development in all genotypes (Figure 11A,B). These data agree with previous studies performed in citrus [31] and tomato fruits [57]. All genotypes peaked in July, with statistically significant values with respect to the other sampling events (Spreadsheet S1). In particular, the expression level in NH was higher (224 mRNA fold increase) than that in other genotypes (32.72, 21.39, and 8.39 mRNA fold increase in LL, TM, and TDV, respectively) (Figure 11A).

The functional role of AO is still unclear, but many hypotheses have been formulated. The high expression values registered at the beginning of fruit development can lead to different hypotheses. (1) A negative feedback process could take part when excessive AsA production occurs. This assumption is supported by the very high and statistically significant correlation reported between the AsA content and the *AO* transcription levels (Table 1). Nevertheless, this speculation could be unlikely, because genotypes with a very high expression value of *AO* (such as NH) do not seem to be affected by its degradation effect. (2) On the other hand, the involvement of AO in cell expansion is still accepted [78], as well as its participation in cell growth [79,80]. This latter assumption could also be compatible with the high activity of AO, mostly in the first part of fruit development. (3) It was also suggested that AO could be involved in oxygen management [81,82]. As found by De Tullio and co-workers [78], low *AO* transcript levels could relate to the last part of the ethylene biosynthetic pathway, which is known to specifically require AsA. The ethylene produced during stage III of citrus fruit might be associated with a lack of *AO* expression, starting in December.

**Figure 12:**
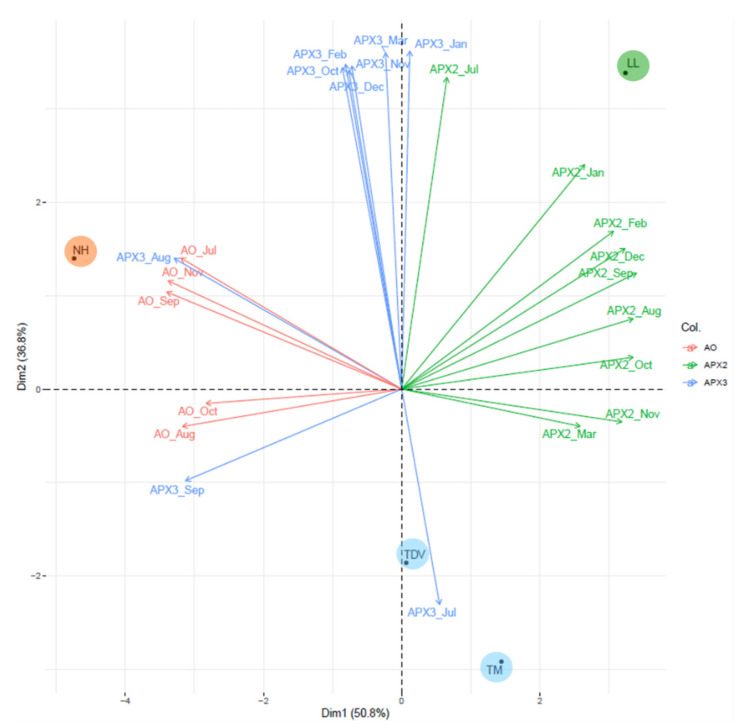
PCA of the expression data of *APX2* and *APX3* isoforms and the *AO* gene evaluated in the juice of Lanelate (LL), Tarocco Meli (TM), Newhall (NH), and Tarocco TDV (TDV) genotypes from July to March. Genes are indicated with different coloured lines. Varieties are indicated in coloured circles.

The PCA, performed using the expression data of this pathway, showed that 87.6% of the variability was derived from two principal components (Dim1 = 50.8%; Dim2 = 36.8%). Dim1 was positively correlated with high levels of *APX2* expression (all samples except July) and was negatively correlated with the expression of *AO* (from July to November) and *APX3* (August and September), separating NH from the other genotypes (Figure 12).

On the other hand, a large group of genes contributed to separate navel (NH and LL) and pigmented oranges (TDV and TM). This separation can be observed in Dim2, in which *APX3* (October, November, December, January, February, and March) and *APX2* (July) were positively correlated with this dimension. On the contrary, the elevated *APX3* (July) value (negatively correlated) would explain the placing of TM in this group, whereas the allocation of TDV in the same group can be explained especially by the low values of the *APX3* (October, November, December, January, February, and March) that were distributed on the opposite side of the quadrant.

**Figure 13:**
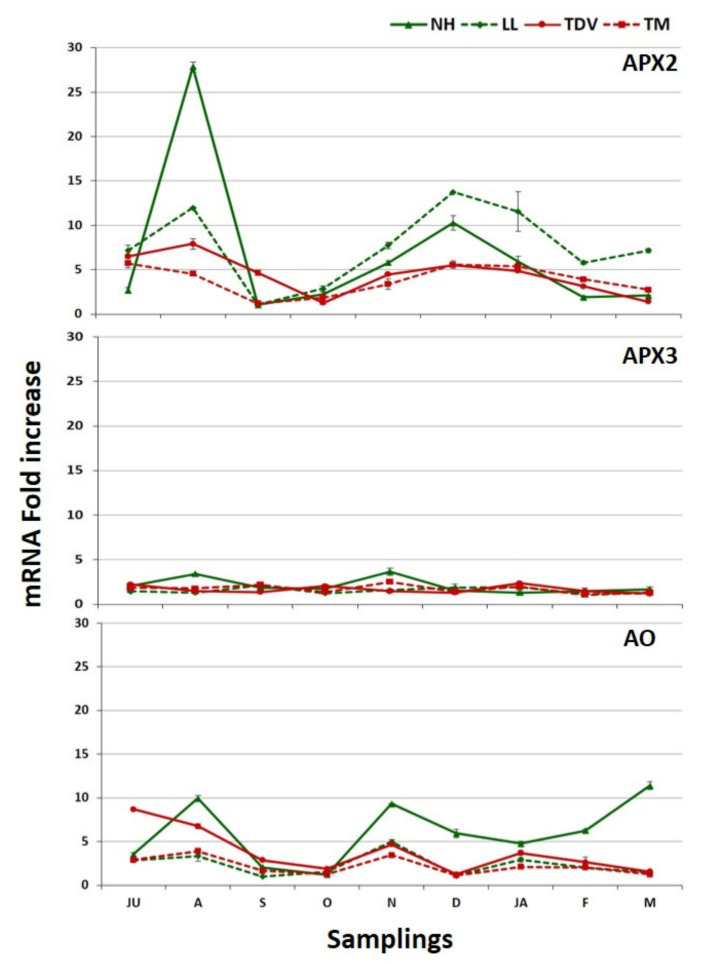
Real-time expression data of *APX2*, *APX3*, and *AO* genes involved in the degradation pathway in the mature leaves of Newhall (NH), Lanelate (LL), Tarocco TDV (TDV), and Tarocco Meli (TM) genotypes from July to March. The data are the mean ± S.E. of at least three replicates.

In the leaves, different expression profiles of the degradation pathway genes were found compared to the juice (Figure 11A and Figure 13). In the leaves, *AO* and *APX3* showed constant expression, while *APX2* displayed higher transcriptional activity (Figure 13). The expression of *APX2* was considerably higher in NH compared to the other genotypes in August (27.81 mRNA fold increase). From November to January, navel genotypes exhibited higher expression values than pigmented ones. On the contrary, the lowest transcription profiles were observed in September/October sampling events for almost all genotypes (Figure 13).

Comparing the expression trend of *APX2*, involved in degradation, and genes involved in recycling (*MDHAR* and *DHAR3*), we observed that the expression of *APX2* (in particular, during winter sampling) was higher. We hypothesize that this enzyme, together with MDHAR and DHAR3, could be critical in citrus leaves for maintaining a higher redox state of AsA, confirming previous studies [19,31]. Furthermore, ascorbate, in its photoprotective role (assumed by APX), repairs light-induced oxidative damage in leaves during the hot season, helping to explain the high expression of *APX2* during the summer and supporting previous evidence [45,83,84].

The expression values of *APX3* in leaves were very low for all genotypes compared to other genes in the degradation pathway, and lower than in juice (Figure 11A and Figure 13). This leads us to conclude that the *APX3* isoform is minimally involved in degradation and the maintenance of the balance of AsA in citrus, confirming a previous report [19].

## 3. Materials and Methods

### 3.1. Plant Material

Four clonal selections of sweet orange, differing in terms of the presence/absence of anthocyanin and maturity time, were selected, namely, “Tarocco TDV” (TDV) (pigmented), “Tarocco Meli” (TM) (pigmented), “Newhall” (NH) (navel), and “Lanelate” (LL) (navel). The choice was based on previous information regarding the major pomological traits [85,86]. Three biological replicates of each genotype, grown using standard cultural practices, were collected from the Experimental Farm of CREA, Research Centre for Olive, Fruit and Citrus Crops (Palazzelli (SR), Italy, 37°20′20.91″ N; 14°53′34.46″ W) and used for all the analyses.

Samples of fruit and mature leaves were collected monthly from July to March. The phases of fruit growth and development were adopted according to Bain [47] (cell division (stage I—April to June), cell enlargement (stage II—June to January), and cell maturation (stage III—January to April)). For all the sampling times and all the biological replicates, 20 fruits were randomly collected from the inner and outer canopy. The leaf samples to be analysed consisted of mature leaves attached near the sampled fruit. Fruits were processed under sterile conditions, and the filtered juice was stored at −80 °C until it was used for chemical and gene expression analyses. Leaves were homogenized at 30 rpm for 30–40 s (TissueLyser II, Qiagen, Hilden, Germany) and stored at −80 °C until the extraction of RNAs for expression analysis.

### 3.2. RNA Extraction, cDNA Synthesis, and Quantitative Real-Time PCR Expression Analysis

Total RNA from juice was extracted according to Bernardi et al. [87]. RNA from leaves was extracted using an SV Total RNA Isolation System (Promega, Madison, WI, USA). The total RNA amount was measured by a Nanodrop 1000 spectrophotometer (Thermo Fisher Scientific, Waltham, MA, USA) and the quality was verified by gel electrophoresis (agarose 0.8% in TAE 1×). cDNA was synthesized using 1 μg of total RNA DNAse free according to the manufacturer’s instructions (High-Capacity cDNA Reverse Transcription Kit, Applied Biosystems, Foster City, CA, USA). One hundred nanograms of cDNA were used for PCR reactions in a 15 µL final volume.

Expression analysis was performed through quantitative real-time PCR, which was conducted using a 7300 Real-Time PCR System (Applied Biosystems, Foster City, CA, USA), with SYBR^®^ Master Mix (Applied Biosystems, Foster City, CA, USA), according to the protocol provided by the manufacturer.

The expression of 17 genes involved in AsA biosynthesis, recycling, and degradation was evaluated in juice and leaves (Table S2). The elongation factor 1α (EF, AY498567) was used as a housekeeping gene [88]. Relative quantification was normalized to the EF reference gene, and the mRNA fold increase was calculated using the standard curve method.

### 3.3. Gene Selection and Primer Design

All genes were selected from previous papers published on citrus [19,31,44] or after a search in the NCBI database [89]; in all cases, we used the *Citrus sinensis* Annotation Project [44] to find the corresponding “gene_id” in the genome. Specific primers were designed with PRIMER3 software (Boston, MA, USA) [90]; when possible, at least one forward and/or reverse primer was designed to straddle exons; in this way, we could be sure that we specifically evaluated the expression of the mRNA, instead of possible residues of DNA. Gene ID, primer sequences, and qRT-PCR product sizes are listed in Table S2.

### 3.4. Chemicals and Physicochemical Analyses

Ascorbic acid was purchased from Sigma Chemical Co. (St. Louis, MO, USA). All other chemicals were of analytical grade, and the solvents used for chromatography were HPLC grade (Merck KGaA, Darmstadt, Germany).

Determination of total soluble solids (TSS), acidity, pH, ripening ratio (TSS/TA), and reduced ascorbic acid (AsA) was performed on fruit juice (Table 2). Juice was extracted from the fruits using an electric citrus squeezer. Titratable acidity (TA), TSS, and pH were determined according to conventional methods [91]. The maturity index was calculated as the ratio between TSS (expressed in °Brix) and TA (expressed as % of citric acid).

#### 3.4.1. Determination of Ascorbic Acid in Juice

The concentration of reduced ascorbic acid was evaluated by liquid chromatography using a Waters Alliance 2695 HPLC device (Waters Corporation, Milford, MA, USA) equipped with a Waters 996 photodiode array detector and Empower software (Waters Corporation, Milford, MA, USA). Briefly, 5 mL of centrifuged juice was poured into a flask and brought up to a total volume of 50 mL with 3% metaphosphoric acid solution. An aliquot of the solution was filtered (0.45-μm filter), and then 20 μL was injected into the HPLC. The mobile phase was 0.02 M H_3_PO_4_, and the detector was set to 260 nm. The quantification of ascorbic acid was performed using a calibration curve and the standard pure compound, as reported by Rapisarda and Intelisano [40]. The results are expressed in mg 100 mL^−1^.

#### 3.4.2. Determination of Ascorbic Acid in Leaves

The concentration of reduced ascorbic acid was evaluated by liquid chromatography using a Waters Alliance 2695 HPLC device (Waters Corporation) equipped with a Waters 996 photodiode array detector and Empower software. The method of extraction was adapted from Zhong and co-workers [92]. More specifically, frozen leaves were ground and powdered using a mortar and pestle chilled with liquid nitrogen. An aliquot of powdered leaves was extracted with 3% metaphosphoric acid solution in the dark under tilted shaking for 4 h, after which the solution was centrifuged. A second extraction of the residue was performed for 2 h in the dark under tilted shaking, followed by centrifugation. Subsequently, the filtrates of the two extractions were combined. An aliquot of the solution was filtered (0.45-μm filter), and then 20 μL was injected into the HPLC. The mobile phase was 0.02 M H_3_PO_4_, and the detector was set to 260 nm. The quantification of ascorbic acid was performed using a calibration curve with a standard pure compound. Results were expressed as mg 100 g FW^−1^. For the validation of the method, the linearity, limit of detection (LOD), and limit of quantitation (LOQ) were determined. Linearity was checked for between 5 mg 100 g^−1^ and 250 mg 100 g^−1^. The standard compound was analysed in triplicate, and a linear calibration curve was constructed from the average values. The linearity of the calibration curve was evaluated by linear regression analysis with a minimally acceptable correlation coefficient (r^2^) of 0.990. LOD and LOQ were calculated based on the standard deviation of the response and the slope. LOD was calculated as 3σ (low concentration)/slope of the calibration curve, while LOQ was calculated as 10σ (low concentration)/slope of the calibration curve. The precision of the methods was expressed as the repeatability relative standard deviation (RSDr) calculated from five independent replicate measurements.

### 3.5. Statistical Analysis

A statistical analysis of the results was performed using the STATSOFT 6.0 program (Vigonza, Padova, Italy). The significant differences (*p* ≤ 0.05; *p* ≤ 0.01) were evaluated by analysis of variance (ANOVA), and mean separation was conducted using a Tukey post hoc test. The Pearson correlation coefficient was used to correlate the AsA content with the relative gene expression (*p* ≤ 0.05).

Principal component analysis (PCA) and heatmaps were computed using R software (R core team) (R Foundation for Statistical Computing, Vienna, Austria). PCAs were calculated using the ‘prcomp’ function of the ‘stat’ R package; outcome was displayed through the ‘factoextra’ package [93]. Heatmaps were generated employing the packages ‘ggplot2′ and ‘Deducer’ [94,95] and the ‘cor’ function of the ‘stat’ R package.

## 4. Conclusions

This is the first work to investigate the citrus AsA metabolism, comparing the fruits and leaves of pigmented and anthocyanin-rich oranges at different maturity times, associating pomological parameters with AsA quantification, and evaluating the expression of the main genes involved in the complex metabolism of vitamin C.

The differences in AsA accumulation in juice and leaves suggest that diverse mechanisms involved in AsA metabolism may act similarly or differently in varying plant tissues, indicating probable tissue specificity, as already described [65].

In the juice, the accumulation of vitamin C was higher during stage II; then, it declined during fruit growth, suggesting major biosynthesis at the beginning of fruit development and a subsequent AsA decrease due to a dilution effect. On the other hand, major accumulation of AsA in the leaves was detected during December and January, particularly in the navel varieties.

The plasticity and the different contribution of various pathways involved in AsA biosynthesis may be closely related to the different stages of development and maturation of fruits and the availability of pathway substrates.

In juice, the *GME* and *GDH* genes appear to play a key role in the l-galactose pathway, being especially expressed during stage II of fruit development. Likewise, in the leaves, the l-galactose pathway plays a major role compared to the other routes, assigning a relevant role in AsA metabolism to *GME* and *GGP*.

The involvement of the myo-inositol pathway likely contributed to the accumulation of AsA in the first part of fruit development and seems to act synergistically with l-galactose, especially during the early developmental stage.

Moreover, we hypothesize that, at the end of stage II and the beginning of stage III, the maintenance of AsA levels could be ascribed to the *GulLO* transcriptional activity, especially in the anthocyanin rich varieties.

Among the analysed *GalUR* isoforms, only *GalUR12* showed involvement in the biosynthesis of AsA in the juice, probably also contributing to the difference between the accumulation of AsA in NH and in the other varieties. On the contrary, in the leaves, a higher expression of all analysed *GalUR* isoforms (especially from October to January) could explain the synergistic contribution of the l-galactose and galacturonate pathways.

To conclude, the pattern of the AsA content in the juice observed in all genotypes may be related to a decrease in the transcript levels of the *GME*, *GDH*, *MyoOx*, and *GalUR12* genes during the sampling events. However, the difference in the accumulation of AsA in the juice between the NH and the other varieties seems to be attributable to differences in the abundance of transcripts and/or correlation relationships of the *GLDH*, *GalUR12*, *APX2*, and *DHAR3* genes.

The main novelty of this study is represented by the higher quantity of vitamin C accumulated in mature leaves of navel genotypes compared to pigmented ones, even though this needs to be further investigated.

Moreover, several post-transcriptional and even post-translational mechanisms are known to be involved in AsA synthesis and in its catabolism. Therefore, other studies need to be performed to evaluate these aspects in our sample collection.

The obtained results could be useful in the further study of the metabolism of vitamin C in citrus. The study of AsA transport mechanisms from leaves to fruit will better clarify the contribution of each pathway to the biosynthesis and accumulation of AsA in citrus.

## Figures and Tables

**Table 1:** Correlation between gene expression and AsA content in the juice and leaves of Lanelate (LL), Tarocco Meli (TM), Newhall (NH), and Tarocco TDV (TDV). Statistically significant correlations (*p* ≤ 0.05) are highlighted in bold.

	Juice				Leaves			
	NH	LL	TDV	TM	NH	LL	TDV	TM
*GMP*	−0.22	**−0.55**	−0.33	**−0.58**	−0.12	0.19	−0.12	−0.30
*GME*	**0.52**	−0.11	**0.56**	**0.52**	**−0.68**	−0.29	−0.08	−0.37
*GGP*	−0.30	**−0.64**	−0.03	**−0.59**	**−0.54**	−0.37	−0.35	**−0.58**
*GPP*	−0.04	**−0.54**	−0.18	**−0.52**	**−0.56**	−0.27	0.26	−0.23
*GDH*	**0.76**	**0.84**	**0.73**	**0.66**	**−0.64**	−0.10	0.09	**−0.59**
*GLDH*	**0.56**	0.27	−0.35	−0.02	−0.30	**0.44**	−0.34	−0.14
*GulLO*	**−0.61**	**−0.54**	**−0.65**	**−0.81**	n.d.	n.d.	n.d.	n.d.
*MyoIFS*	0.18	−0.11	−0.04	0.19	**−0.63**	**−0.55**	**−0.45**	**−0.43**
*MyoOx*	**0.79**	**0.85**	**0.88**	**0.80**	0.05	−0.16	−0.28	0.37
*MDHAR*	**−0.46**	**−0.67**	0.38	**−0.84**	**−0.59**	0.10	−0.25	**−0.42**
*DHAR*	**0.58**	**−0.50**	**0.49**	−0.03	**−0.59**	−0.22	**−0.57**	−0.30
*GalUR8*	**−0.75**	**−0.60**	**−0.65**	**−0.56**	−0.34	**0.47**	**−0.58**	−0.25
*GalUR10*	0.23	0.20	0.27	0.33	**−0.58**	**0.68**	0.23	0.08
*GalUR12*	**0.74**	0.16	0.11	**0.56**	**−0.52**	−0.24	**−0.71**	**−0.61**
*APX2*	0.03	**−0.51**	−0.07	−0.11	−0.32	**0.45**	0.37	0.02
*APX3*	−0.31	**−0.49**	−0.17	0.03	**−0.75**	**0.50**	0.16	−0.27
*AO*	**0.90**	**0.90**	**0.93**	**0.84**	−0.11	−0.35	0.26	**−0.46**

**Table 2:** Analysis of five parameters measured in navel and pigmented oranges. Changes in TSS, TA, TSS/TA ratio, and AsA (mg/100 mL) in fruits of Newhall (NH), Lanelate (LL), Tarocco TDV (TDV), and Tarocco Meli (TM).

	TA	pH	TSS	TSS/TA	AsA
*August*					
**NH**	3.07	2.88	11.26	3.59	89.20
**LL**	4.35	2.74	11.03	2.53	85.00
**TDV**	4.48	2.76	10.83	2.42	88.14
**TM**	5.99	2.63	10.56	1.76	80.18
*September*					
**NH**	2.32	3.07	12.21	5.26	79.45
**LL**	3.36	2.92	10.20	3.03	68.31
**TDV**	3.87	2.93	9.60	2.48	69.37
**TM**	5.19	2.72	10.00	1.94	64.87
*October*					
**NH**	2.46	3.16	11.22	4.56	61.70
**LL**	2.62	3.23	8.85	3.39	54.99
**TDV**	3.15	3.13	9.46	3.00	57.90
**TM**	4.48	2.97	9.21	2.06	65.16
*November*					
**NH**	1.54	3.15	11.83	7.76	67.93
**LL**	1.77	3.06	8.57	4.84	56.89
**TDV**	2.02	3.01	9.45	4.69	61.74
**TM**	3.19	2.76	9.24	2.90	61.50
*December*					
**NH**	1.25	3.38	12.01	9.74	61.81
**LL**	1.49	3.24	9.47	6.39	47.03
**TDV**	1.61	3.18	9.61	6.00	53.76
**TM**	2.70	2.86	9.34	3.47	46.57
*January*					
**NH**	1.13	3.38	12.17	10.87	62.12
**LL**	1.45	3.27	9.64	6.83	47.95
**TDV**	1.75	3.24	9.64	5.52	50.55
**TM**	2.72	2.84	9.30	3.42	49.92
*February*					
**NH**	0.86	3.55	13.04	16.58	60.18
**LL**	1.04	3.41	10.61	10.26	53.10
**TDV**	0.98	3.51	10.21	10.43	53.38
**TM**	2.02	2.99	10.59	6.60	52.98
*March*					
**NH**	0.88	3.63	12.00	14.06	60.40
**LL**	0.96	3.56	10.50	10.96	47.63
**TDV**	1.11	3.47	9.78	8.81	51.63
**TM**	1.62	2.96	10.22	5.09	50.71

## Supplementary Materials

The following are available online at https://www.mdpi.com/article/10.3390/plants10071291/s1, Figure S1: Real-time expression data of the GalUR8, GalUR10, and GalUR12 genes in (A) the mature leaves and (B) juice of Lanelate (LL), Tarocco Meli (TM), Newhall (NH), and Tarocco TDV (TDV) genotypes during sampling from July to March. The data are the mean ± S.E. of at least three replicates, Figure S2: Real-time expression data of DHAR3 and MDHAR in (A) the mature leaves and (B) juice of Lanelate (LL), Tarocco Meli (TM), Newhall (NH), and Tarocco TDV (TDV) genotypes during sampling from July to March. The data are the mean ± S.E. of at least three replicates, Table S1: Correlation between the AsA content, diameter, and weight of Lanelate (LL), Tarocco Meli (TM), Newhall (NH), and Tarocco TDV (TDV) fruits. Pearson’s correlation coefficient (during sampling) between the AsA content and equatorial and longitudinal diameter ratio as well as fruit weight, Table S2: Gene ID, primer sequences, and real-time PCR amplicon sizes, Spreadsheet S1: One-way analysis of variance (ANOVA) performed on data collected for the four genotypes (Lanelate (LL), Tarocco Meli (TM), Newhall (NH), and Tarocco TDV (TDV)) using STATISTICA 6.0 software (StatSoft Italia srl, Vigonza, Padova, Italy). Mean partitioning was carried out by Tukey’s HSD test using variety as a categorical factor (for *GulLO*, *GGP*, *GPP*, *GDH*, *APX2*, and *APX3* genes) and sampling time (for *GulLO*, *GDH*, *GalUR10*, and *AO* genes). *p* ≤ 0.01—capital letters, *p* ≤ 0.05—lowercase letters, ns—not significant.
