# Supplementary material for: A Transcriptional Analysis of the Genes Involved in the Ascorbic Acid Pathways Based on a Comparison of the Juice and Leaves of Navel and Anthocyanin-Rich Sweet Orange Varieties
Source: Plants (Basel). 2021 Jun 24;10(7):1291. doi: 10.3390/plants10071291 (PMC8309047; doi:10.3390/plants10071291)
Supplement: Supplementary file 1 [file plants-10-01291-s001.zip › Table S1.pdf]

Table S1

	Correlation with diameters	Correlation with weight
**NH**	-0.97	-0.99
**LL**	-0.93	-0.93
**TDV**	-0.94	-0.95
**TM**	-0.90	-0.93
