# Supplementary material for: A Transcriptional Analysis of the Genes Involved in the Ascorbic Acid Pathways Based on a Comparison of the Juice and Leaves of Navel and Anthocyanin-Rich Sweet Orange Varieties
Source: Plants (Basel). 2021 Jun 24;10(7):1291. doi: 10.3390/plants10071291 (PMC8309047; doi:10.3390/plants10071291)
Supplement: Supplementary file 1 [file plants-10-01291-s001.zip › Table S2.pdf]

Table S2. Genes id, primer sequences and Real-time PCR amplicons size.

Gene name	Gene id	Primer sequence (5'-3')	Amplicon size (bp)
L-galactose pathway[1]			
GDP-mannose pyrophosphorylase (GMP)	Cs7g31960	GMP-Fw-ggtgcatgagagtgctcaaa<br>GMP-Rev-tacagtgcagcgagagagtc	109
GDP-mannose-3,5-epimerase (GME)[2]	Cs3g10840	GME-Fw-gcctccagtgcctgcatct<br>GME-Rev-ggcatcagactccttcaagctt	72
GDP-L-galactose-pyrophosphorylase (GGP)	Cs4g09870	GGP-Fw-agtacgggcatgtgctgttg<br>GGP-Rev-caagaagctatcacggtcaatcc	74
L-galactose-1-phosphate phosphatase (GPP)[3]	Cs2g02090.1	GPP-Fw-tcttggacactgcagttgatg<br>GPP-Rev-tctgtgaccaaatccacctg	80
GDP-L-galactose dehydrogenase (GDH)	Cs8g03180	GDH-Fw-ggcgttccgagaaatgagta<br>GDH-Rev-ggattttccctgcttccttc	229
L-galactono-1,4-lactone dehydrogenase (GLDH)	orange1.1t00763	GLDH-Fw-caccgcctcctcttcaacaa<br>GLDH-Rev-tgagagcgtacggattgaaggt	69
L-gulose pathway			
L-gulonolactone oxidase 6 (GulLO)[4]	XM_006491294.1	GulLO-Fw-aggacccaatgactccaagg<br>GulLO-Rev-ccatacttgaacaccgcgag	73
Myo-inositol pathway[1]			
Inositol oxygenase (MyoOx)	Cs3g02580.1	MyoOx-Fw-cttattcagaaggccgtgga<br>MyoOx-Rev-cccggtgtaaggcatagaaa	150
Inositol-3-phosphate synthase (MyoIPS)	Cs1g01700	MyoIFS-Fw-ctcttttggctgctccaatc<br>MyoIFS-Rev-gttgttctcaggagccaagc	245
Galacturonate pathway[1,5]			
Galacturonic acid reductase8 (GalUR8)	Cs1g07420.1	GalUR8-Fw-tccctccttccatcaatcag<br>GalUR8-Rev-gacattcatgcccttgacct	90
Galacturonic acid reductase10 (GalUR10)	orange1.1t00001.2	GalUR10-Fw-ggagaagcaatcgatgaagc<br>GalUR10-Rev-tgagcgtcattcaaccagag	92
Galacturonic acid reductase12 (GalUR12)	orange1.1t01634.1	GalUR12-Fw-ggtgggtttatcagcaagga<br>GalUR12-Rev-tcggcacttaattcccaatc	100
Recycling pathway[1]			
Monodehydroascorbate reductase (MDHAR)	Cs5g03080	MDHAR-Fw tccctctggtgtcctcagtc<br>MDHAR-Rev-tgagtatgcgacgacaaagc	92
Glutathione S-transferase DHAR3 (DHAR3)	Cs6g17520	DHAR-Fw-ctgagaaggcttcagtcgga<br>DHAR-Rev-atgcctgctcagatccatca	170
Degradation pathway[1]			
Ascorbate oxidase (AO)[3]	Cs2g29090.1	AO-Fw-cgggttcactaagtgggcta<br>AO-Rev-tggaggtttcatcacgtcatac	151
L-ascorbate peroxidase2 (APX2)	Cs6g04140	APX2-Fw-tcagttggctggagttgttg<br>APX2-Rev-tcagatttgtccggtcttcc	81
L-ascorbate peroxidase3 (APX3)	Cs8g02730	APX3-Fw-ttaccggtcgttgacacaga<br>APX3-Rev-acgctagacggagcatgatt	109

[1] All genes were evaluated on juice and leaves.
[2] The gene GME was previously evaluated in Yang et al., 2011.
[3] Primers are the same used in Alos et al., 2014.
[4] The expression of this gene was evaluated only on the juice.
[5] For this pathway, was evaluated three isoforms of the same gene, as described by Xu et al., 2012.
