# Supplementary figures and images for: A Transcriptional Analysis of the Genes Involved in the Ascorbic Acid Pathways Based on a Comparison of the Juice and Leaves of Navel and Anthocyanin-Rich Sweet Orange Varieties
Source: Plants (Basel). 2021 Jun 24;10(7):1291. doi: 10.3390/plants10071291 (PMC8309047; doi:10.3390/plants10071291)

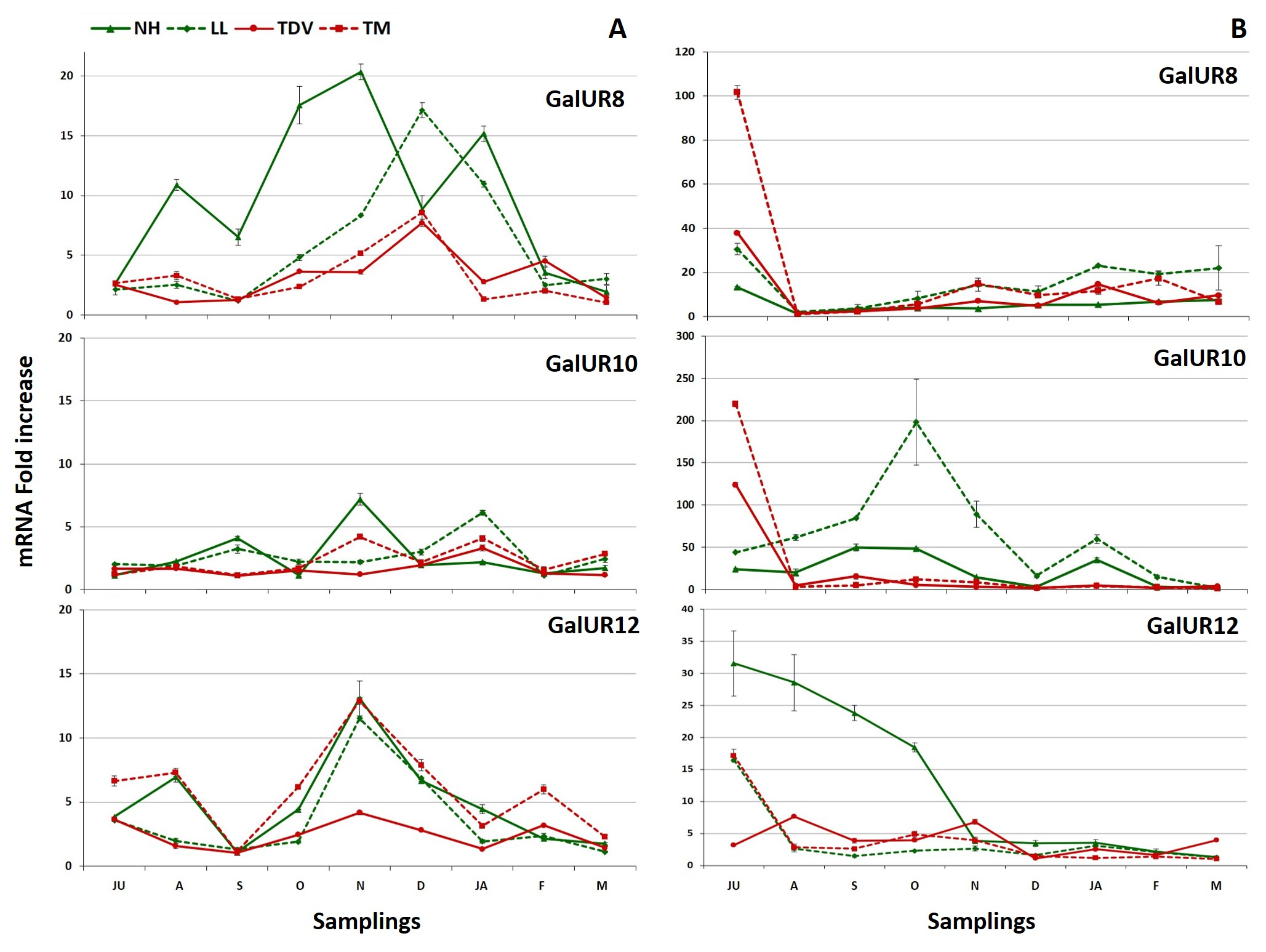

Supplement: Supplementary file 1 [file plants-10-01291-s001.zip › Figure S1.jpg]

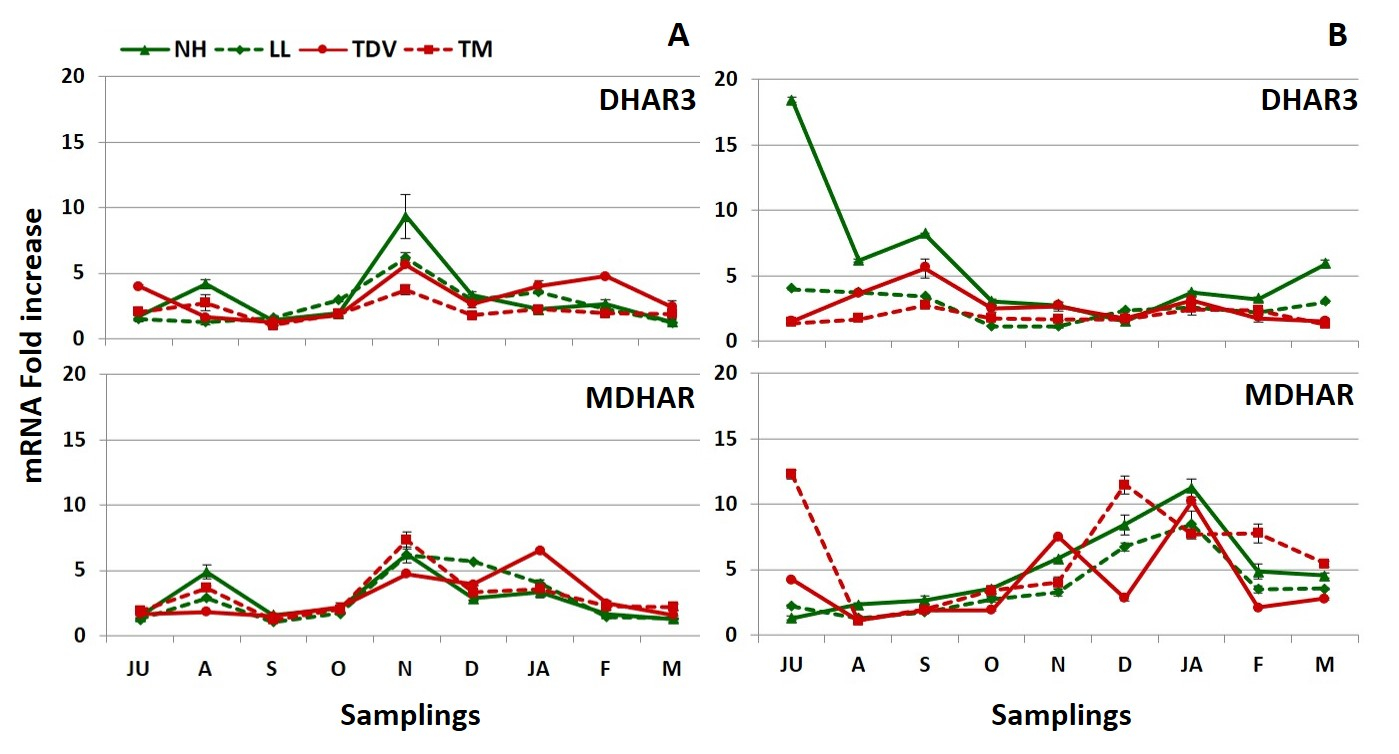

Supplement: Supplementary file 1 [file plants-10-01291-s001.zip › Figure S2.jpg]
